# Elimination of *Mycoplasma* Contamination from Infected Human Hepatocyte C3A Cells by Intraperitoneal Injection in BALB/c Mice

**DOI:** 10.3389/fcimb.2017.00440

**Published:** 2017-10-12

**Authors:** Jun Weng, Yang Li, Lei Cai, Ting Li, Gongze Peng, Chaoyi Fu, Xu Han, Haiyan Li, Zesheng Jiang, Zhi Zhang, Jiang Du, Qing Peng, Yi Gao

**Affiliations:** ^1^Department of Hepatobiliary Surgery II, Zhujiang Hospital, Southern Medical University, Guangzhou, China; ^2^Artificial Organs and Tissue Engineering Centre of Guangdong Province, Guangzhou, China; ^3^Department of Pharmacology, Shantou University Medical College, Shantou, China; ^4^Department of Pediatrics, Zhujiang Hospital, Southern Medical University, Guangzhou, China; ^5^State Key Laboratory of Organ Failure Research, Southern Medical University, Guangzhou, China

**Keywords:** *Mycoplasma*, elimination, intraperitoneal inoculation, monoclonal cells, cell cross-contamination

## Abstract

**Background/Aims:** The use of antibiotics to eliminate *Mycoplasma* contamination has some serious limitations. *Mycoplasma* contamination can be eliminated by intraperitoneal injection of BALB/c mice with contaminated cells combined with screening monoclonal cells. However, *in vivo* passage in mice after injection with contaminated cells requires a long duration (20–54 days). Furthermore, it is important to monitor for cross-contamination of mouse and human cells, xenotropic murine leukemia virus-related virus (XMRV) infection, and altered cell function after the *in vivo* treatment. The present study aimed to validate a reliable and simplified method to eliminate mycoplasma contamination from human hepatocytes. BALB/c mice were injected with paraffin oil prior to injection with cells, in order to shorten duration of intraperitoneal passage. Cross-contamination of mouse and human cells, XMRV infection and cell function-related genes and proteins were also evaluated.

**Methods:** PCR and DNA sequencing were used to confirm *Mycoplasma hyorhinis* (*M. hyorhinis*) contamination in human hepatocyte C3A cells. Five BALB/c mice were intraperitoneally injected with 0.5 ml paraffin oil 1 week before injection of the cells. The mice were then intraperitoneally injected with C3A hepatocytes (5.0 × 10^6^/ml) contaminated with *M. hyorhinis* (6.2 ± 2.2 × 10^8^ CFU/ml). Ascites were collected for monoclonal cell screening on the 14th day after injection of contaminated cells. Elimination of mycoplasma from cells was determined by PCR and Transmission Electron Microscopy (TEM). Human–mouse cell and XMRV contamination were also detected by PCR. Quantitative reverse transcription PCR and western blotting were used to compare the expression of genes and proteins among treated cells, non-treated infected cells, and uninfected cells.

**Results:** Fourteen days after injection with cells, 4 of the 5 mice had ascites. Hepatocyte colonies extracted from the ascites of four mice were all mycoplasma-free. There was no cell cross-contamination or XMRV infection in treated cell cultures. Elimination of *Mycoplasma* resulted in partial or complete recovery in the expression of ALB, TF, and CYP3A4 genes as well as proteins. Proliferation of the treated cells was not significantly affected by this management.

**Conclusion:** The method of elimination of *Mycoplasma* contamination in this study was validated and reproducible. Success was achieved in four of five cases examined. Compared to the previous studies, the duration of intraperitoneal passage in this study was significantly shorter.

## Introduction

*Mycoplasma* contamination of cultured cells poses a serious challenge to biological and biopharmaceutical studies, since infection rates of cell cultures can range from 15 to 100% (Kazemiha et al., 2016). Although a number of methods have been evaluated to eliminate *Mycoplasma* contamination, treatment of cell cultures with antibiotics remains the most widely used because it is simple and rapid (Drexler and Uphoff, 2002; Hopfe et al., 2013). However, using antibiotics to eliminate *Mycoplasma* contamination has some serious limitations. Some bacteriostatic antimicrobial agents inhibit *Mycoplasma* growth without completely eradicating the contaminant (Lincoln and Gabridge, 1998), while some anti-*Mycoplasma* antibiotics have no effect because of the development of antibiotic-resistant *Mycoplasma* (Drexler and Uphoff, 2002). Additionally, although some antibiotics, such as aminoglycosides and lincosamides are effective at eradicating *Mycoplasma* contamination, they are cytotoxic to the cultured cells (Drexler and Uphoff, 2002; Laleh Nikfarjam, 2012). Recent data also suggested that some anti-*Mycoplasma* antibiotics are mostly effective in the extracellular media and not as much against intracellular *Mycoplasma* (Degeling et al., 2012).

Alternative ways to effectively eliminate *Mycoplasma* contamination in cell cultures include co-cultivating contaminated cells with primary human or mouse macrophages *in vitro* or by passaging contaminated cells in mice (Schimmelpfeng et al., 1980; Howell et al., 1982; Lombardo and Lanks, 1982; Roseto et al., 1984; Carroll and O'Kennedy, 1988; Hirschberg et al., 1989). In addition to the fact that acquisition of human macrophages is an expensive and demanding procedure, techniques for co-culture of contaminated cells with human or mice macrophages *in vitro* are not well-standardized. *In vivo* strategies whereby BALB/c mice are intraperitoneally injected with contaminated cells may therefore be the most effective mean of eliminating *Mycoplasma* contamination.

The major concerns and challenges of *in vivo* passage of cells in mice include (1) long duration (20–54 days) of *in vivo* passage (Lombardo and Lanks, 1982); (2) the possibility of cross-contamination of mouse and human cells (Schimmelpfeng et al., 1980); (3) changes in cell function (e.g., proliferation, gene expression and protein expression) after *in vivo* treatment; (4) the possibility of changes in cell characteristics such as short tandem repeats (STR), (5) the possibility that intracellular *Mycoplasma* cannot be cleared by *in vivo* treatment; and (6) the risk of infection with xenotropic murine leukemia virus-related virus (XMRV) (Naseer et al., 2015).

In this study, we evaluated a method to eliminate *Mycoplasma hyorhinis* (*M*. *hyorhinis*) contamination from C3A human hepatocytes without antibiotic treatment. Our technique included injection of normal BALB/c mice with paraffin oil prior to injection with cells in order to shorten the duration of *in vivo* passage. We validated the effectiveness of this strategy by continuous PCR, Transmission Electron Microscopy (TEM) and Hoechst 33258 staining.

## Materials and methods

### Cell cultures

Mycoplasma-free human hepatocyte C3A cells, a clonal derivative of Hep G2 cells with high albumin (ALB) production, were obtained from American Type Culture Collection (ATCC). Before the *in vivo* experiment, continuous PCR and DNA sequencing confirmed that C3A used in this study had been contaminated by Mycoplasma for more than 1 month. Cells were grown to adherence in standard plastic plates and flasks (Biofil, Guangzhou, China) in Eagle's minimum essential medium (EMEM) (ATCC Lot:63609149) supplemented with 20% *Mycoplasma*-free, heat-inactivated fetal bovine serum (FBS) (Gibco, Lot:42F5364K). Cultures were maintained at 37°C, in the presence of 5% CO_2_ and 90% humidity. No other supplements (such as antibiotics) besides FBS were added to the culture medium. Mycoplasma-infected and uninfected cells were cultured in separate incubators in order to avoid cross-contamination. Cells were thawed and cultured for 6 days before being used for elimination experiments. The cured cells which were harvested at Day 49 were designated as Tc. Non-treated contaminated cells cultured for 6 days after being thawed were designated as 1d M(+) cells, and non-infected cells were designated as M(−) cells. 1d M(+) cells which were further cultured *in vitro* for 43 days were designated as 43d M(+) cells.

### Mycoplasma detection and identification

Cell culture supernatants were collected every 7 days from the different experimental groups and stored in sterile DNA-free microcentrifuge tubes at −20°C. *Mycoplasma* contamination was detected by PCR using primers specifically designed toward a highly conserved 16S rRNA coding region of the *Mycoplasma* genome that can be used to identify the most common infecting species (Huada, Shenzhen, China) Primer sequences are listed in Table 1. Cell culture supernatants were thawed on ice and directly used for PCR. The PCR reaction mixture contained 5 μL of culture supernatant, 0.5 μL each of forward and reverse primers, 10 μL of 2 × PCR Master mix (Toyobo, Osaka, Japan, Cat: KOD 401) and 4 μL deionized water (ddH_2_O). The positive control reaction contained 2 μL of DNA sample from the *M. hyorhinis* genome (>10^4^ copies, Primerdesign, England), and the negative control contained 2 μL of deionized water instead of the sample. Cycling conditions included an initial denaturation step for 5 min at 95°C, followed by 35 cycles of 94°C for 1 min, 57°C for 1 min, and 72°C for 1 min. The final elongation was done at 72°C for 6 min. PCR reaction products (5 μL) were analyzed on 1.4% agarose gels. Mycoplasma species were identified by subjecting PCR reaction products to bidirectional sequencing by The Beijing Genomics Institute (Shenzhen, China). All DNA sequence data were blast-searched against the NCBI Nucleotide database.

**Table 1:** Primers for PCR and qRT-PCR.

**Gene**	**Forward primer**	**Reverse primer**
*Mycoplasma*	5′-TCGTAACAAGGTATCCCTAC-3′	5′-GCATCCACCAAATACTCT-3′
Human COX-I	5′-TAGACATCGTACTACACGACACG-3′	5′-TCCAGGTTTATGGAGGGTTC-3′
Mice COX-I	5′-ATTACAGCCGTACGCTCCTAT-3′	5′-CCCAAAGAATCAGAACAGATC-3′
XMRV	5′-CTGGATCTATTGATTTGAGTTGG-3′	5′-GCTTTATTGGGAACACGGGTA-3′
ALB	5′-GCCCTGTGCAGAAGACTATC-3′	5′-GGGAACGTATGTTTCATCGA-3′
TF	5′-TGAATGCAAGCCTGTGAAGT-3′	5′-TAGACAAACCCTCCATCCAA-3′
CPS1	5′-AAGGATGCTACCCGGAAGA-3′	5′-CAATGAAGTCAACCCCAAGA-3′
CYP3A4	5′-AAAGTCGCCTCGAAGATACA-3′	5′-GAGAACACTGCTCGTGGTT-3′
CYP2D6	5′-ACCACTGCCGTGATTCATG-3′	5′-GGTTGGTGATGAGTGTCGTT-3′
β-actin	5′-TGGATCAGCAAGCAGGAGTA-3′	5′-TCGGCCACATTGTGAACTTT-3′
Human GAPDH	5′-AGAAGGCTGGGGCTCATTTG-3′	5′-AGGGGCCATCCACAGTCTTC-3′
Mice GAPDH	5′-GGCCTCCAAGGAGTAAGAAA-3′	5′-GCCCCTCCTGTTATTATGG-3′

### Transmission electron microscopy (TEM)

Tc, 1d M(+) cell, and 43d M(+) cell were collected for TEM analysis and to confirm the PCR results. Cells were fixed for 12 h in a solution of 2% glutaraldehyde in 0.1 M PBS, and then exposed to a solution of 2% osmium tetraoxide in 0.1 M PBS for 1 h. The samples were dehydrated in ethanol, infiltrated with a mixture of ethanol and epoxy resin (1:1) for 1 h and polymerized with pure epoxy resin at 60°C for 18 h. Ultra-thin sections (90 nm), were mounted on 200-mesh thin-bar copper grids (Agar) and stained with uranyl acetate and Reynold's stain. Sections were subjected to TEM (H-7500, Hitchi, Japan) at an accelerating voltage of 60 Kv.

### Determination of colony-forming units (CFU) of mycoplasma of cell suspensions

Contaminated cell cultures were centrifuged and the pellets were resuspended with PBS. Suspensions of *Mycoplasma*-contaminated cells (4 × 10^6^ cells/ml) were serially diluted and 0.1 ml of each dilution was inoculated on Mycoplasma agar base (Senbeijia, SBJ-ME-1274, China) supplemented with 10% horse serum (Gibco, 26050070, New Zealand) and 100 U/ml penicillin. The agar plates were then incubated at 37°C for 7 days. Colonies on agar plates were stained with Dienes (Senbeijia, SBJ-1744, China) for *Mycoplasma* counting.

### Animals

Adult female BALB/c mice (8–10 weeks old) were purchased from the Southern Medical University Animal Laboratories. Mice were fed standard forage and housed in cages within sound-attenuated, temperature-controlled isolation chambers with a 12:12 light-dark cycle (lights on at 4 a.m.) at 22°C ambient temperature. To avoid any chance of cross-contamination of *Mycoplasma* between mice, mice were individually housed in cages. All animal work was conducted in compliance with the recommendations in the Guide for the Care and Use of Laboratory Animals. All animal protocols were approved by Animal Ethics Committee of the Southern Medical University, and all efforts were made to minimize suffering of mice.

### Mycoplasma elimination

**Figure 1:**
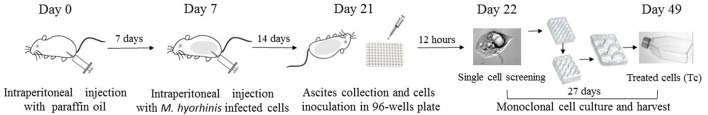
Flowchart of *Mycoplasma* elimination. Aseptic inflammation was induced in BALB/c mice by intraperitoneal injection (i.p) with 0.5 ml sterile paraffin oil 7 days before cells injection. 1d M(+) cell were intraperitoneal injected with a 0.5 ml of cell suspension (4 × 10^6^/ml) in mice in day 7. Cells from the ascites were collected and inoculated in 96-well plate in day 21. After 12 h, the single attached cells were screened by microscopy and cultured for 27 days. Cells were harvested in 25-cm^2^ culture flasks in day 49 for further experiments.

Aseptic inflammation was induced in BALB/c mice (*n* = 5) by intraperitoneal injection (i.p) of 0.5 ml sterile paraffin oil (CAS#: 8042-47-5, DAMAO, China) 7 days before the C3A cells injection. 1d M(+) C3A cells were digested with 0.25% trypsin (Gibco, Hangzhou, China, Cat: 25200), centrifuged at 1,200 rpm for 3 min, then resuspended in normal saline at a concentration of 4 × 10^6^ cells /ml. Each mouse was intraperitoneally injected with a 0.5 ml of C3A cell suspension and the mice were sacrificed after 14 days. Ascites were collected using a sterile syringe, and centrifuged at 800 rpm for 5 min to harvest the cells. The cells were resuspended in EMEM medium (20% FBS) to obtain a concentration of 10–20 cells/ml. Cells were plated in 96 well plates (150 μL/well) and incubated for 12 h. The single attached cells in plates were screened by microscopy (Axio Observer, Zeiss). The cells were cultured and degested by 0.25% trypsin when cells showed significant contact inhibition, or were more than 70% confluent, and then reseeded to a larger plate or flask (Figure 1). The process of culture and reseeding continued for 27 days and 5 × 10^6^ cells were harvested for further experiments.

### Human–mouse cell contamination testing

Genomic DNA was extracted from Tc, M(−) cell, and control mouse liver AML12 cells (ATCC) using the Wizard Genomic DNA Purification Kit (Promega, Madison, WI, USA, Cat: A1120). The integrity of genomic DNA was evaluated electrophoretically. DNA samples were then diluted 1:20 for PCR assays using human and mouse COX-I primers to determine species cross-contaminations (Parodi et al., 2002; Cooper et al., 2007) (primer sequences listed in Table 1). The PCR reaction mixture (25 μl) contained 0.5 μl DNA, 2.5 μl of 2 mM dNTP mixture, 2.5 μl 10 × KOD buffer, 0.3 μL each of forward and reverse primers, 1.5 μL of 25 mM MgSO_4_, 0.3 μL KOD Plus Neo (Toyobo, Osaka, Japan, Cat: KOD401B), and 17.1 μL ddH_2_O. The cycling conditions included an initial denaturation step of 94°C for 3 min, followed by 30 cycles of 98°C for 15 s, 58°C for 15 s, and 68°C for 30 s. The final elongation was done at 68°C for 5 min. DNA samples from M(−) cell and AML12 cells were used as positive controls, and ddH_2_O was used as a negative control. PCR products (5 μL) were analyzed on 1% agarose gels.

### Mycoplasma detection using immunofluorescence of hoechst 33258 DNA staining

Cells were tested using a *Mycoplasma* detection kit (C0296, Beyotime, Hangzhou, China) according to the manufacturer's instructions. The assay was based on Hoechst 33,258 staining of *Mycoplasma* DNA, which was observed and photographed using a fluorescence microscope (Axio Observer, Zeiss).

### Detection of XMRV (xenotropic murine leukemia virus-related virus) by PCR

XMRV was detected by PCR assays as previously described (Naseer et al., 2015). PCR reaction products were identified on 1% agarose gels.

### Quantitative reverse transcription PCR(qRT-PCR) to determine levels of functional genes in cells

Total RNA was extracted from Tc, 1d M(+) cell, 43d M(+) cell, and M(−) cell cells using Trizol reagent (Invitrogen, Carlsbad, CA, USA, Cat: 15596018) according to the manufacturer's instructions. After reverse transcription, cDNAs were used for qRT-PCR. The reaction mixture (20 μl) contained 5 μl cDNA (1:20 dilution), 0.5 μL each of forward and reverse primers, 10 μl of 2x SYBR Green qRT-PCR SuperMix 10 μl (Invitrogen, Cat:11733038), and 4 μl of RNase-free H_2_O. Cycling conditions included 1 cycle of 50°C for 2 min, 1 cycle of 95°C for 2 min, and 40 cycles of 95°C for 15 s and 60°C for 32 s using an ABI Prism 7500 Sequence Detection System (Application Binary Interface, USA). The primer sequences are listed in Table 1. The mRNA levels were normalized to that of β-actin [ΔCt = ΔCt(gene) − ΔC (β-actin), ΔΔCt = ΔCt (gene in experiment group) − ΔCt (gene in control group) Expression = 2 − ΔΔCt].

### Western blotting to evaluate protein expression

Cell samples were RIPA lysed buffer (P0013E, Beyotime) and protein concentration of the lysates was determined using the BCA kit (P0010S, Beyotime). Protein samples were separated by SDS-PAGE, transferred to PVDF membranes, and blocked in 10% milk in Tris-buffered saline. The membranes were then incubated at 4°C overnight in a 1:500 dilution of anti-human ALB (sc-271605, Santa Cruz), and then incubated in HRP-conjugated secondary antibody with a dilution of 1:3,000 for 2 h (BA1051, BA1055, Boster). Protein bands were visualized with an ECL kit (WBKLS0050, Merck Millipore). Data were analyzed using Image J 3.0. The gray ratio of ALB proteins and β-actin represented the relative level of the target protein. CPS1, TF, CYP3A4, CYP2D6 proteins were similarly detected by western blotting using their specific antibodies with the dilutions of 1:500, 1:10,000, 1:2,000, and 1:1,000, respectively (ab64613, ab82411, ab3572 Abcam, 93867s Cell Signaling Technology).

### Short tandem repeats (STR) identification of Tc and data comparison

STR identification in Tc cells was performed using the PowerPlex16 HS System (Promega Corp, Madison, USA) according to the manufacturer's instructions and as previously described (Ensenberger et al., 2010). The technique uses capillary electrophoresis to detect 16 STR loci. The results were compared to the C3A cell information provided by ATCC.

### MTT assay to detect cell proliferation

Cells from the Tc, M(−) cell, 1d M(+) cell, and 43d M(+) cell groups were inoculated in 96-well plates at a density of 1,000 cells/well (200 μl). MTT solution (Invitrogen, Cat: M6494) at a concentration of 5 mg/ml in 0.1 M PBS was added into each well (20 μl/well). All tests were run in at least five wells. The cells were incubated for 4 h at 37°C in an atmosphere containing 5% CO_2_ prior to the addition of 150 μl of dimethyl sulfoxide (DMSO) into each well. The cells were incubated for 10 min to dissolve formazan crystals, and the absorbance was read at 490 nm using a microplate reader (Bio-tek Elx800). Five wells containing only medium were used as controls.

### Statement of biohazard safety

All the procedures followed the guidelines of Biological Safety Regulations of Pathogenic Microbiology Laboratory of China.

### Statistical method

Data are presented by mean ± standard deviation (SD). Differences among groups were assessed using one-way ANOVA or Kruskal-Wallis non-parametric test for continuous variables. Post-hoc pairwise multiple comparisons were made with LSD or Dunnett's procedure if significant differences were found among groups. *P* < 0.05 was considered statistically significant. Analyses were carried out using SPSS version 22.0.

## Results

### Detection and identification of *M. hyorhinis*

**Figure 2:**
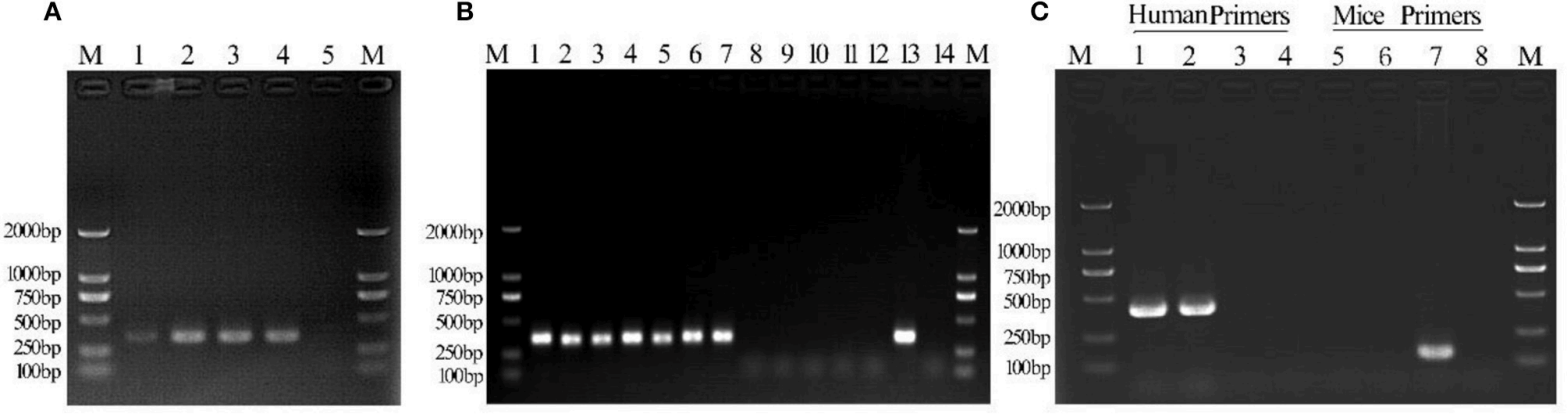
Detection of *Mycoplasma* contamination in infected C3A cells. Lane 1: day 2 culture; lane 2: day 4 culture; lane 3: day 6 culture; lane 4: positive control; lane 5, negative control. **(B)** *Mycoplasma* detection in Tc and M(+) cell groups. Lane 1: 1d M(+) cell; lane 2: 8d M(+) cell; lane 3: 15d M(+) cell; lane 4: 22d M(+) cell; lane 5: 27d M(+) cell; lane 6: 36d M(+) cell; lane 7: 43d M(+) cell; lane 8: 1d Tc; lane 9: 8d Tc; lane 10: 15d Tc; lane 11: 22d Tc; lane 12: 29d Tc; lane 13: positive control; lane 14: negative control. **(C)** Monoclonal human–mice cell contamination. Lane 1: Tc+ human COX-I primers; lane 2: M(−) cell+ human COX-I primers; lane 3: ML12+human COX-I primers; lane 4: ddH_2_0+human COX-I primers; lane 5: Tc+ mice COX-I primers; lane 6: M(−) cell+mice COX-I primers; lane 7: AML12+mice COX-I primers; lane 8: ddH_2_0+mice COX-I primers; lane M, 1-2000-bp DNA marker (Takara).

PCR analysis showed the presence of a 343 bp band in all *Mycoplasma*-infected C3A cells over a duration of 6 days of culture (Figure 2A). DNA sequence analysis of PCR products obtained on days 2, 4, and 6 of culture showed 100% identity with *M. hyorhinis* DBS 1050. Cell suspensions for intraperitoneal injections contained 6.2 ± 2.2 × 10^8^ CFU/ml of *Mycoplasma* (Supplementary Figure 1).

### Monoclonal cell selection

**Figure 3:**
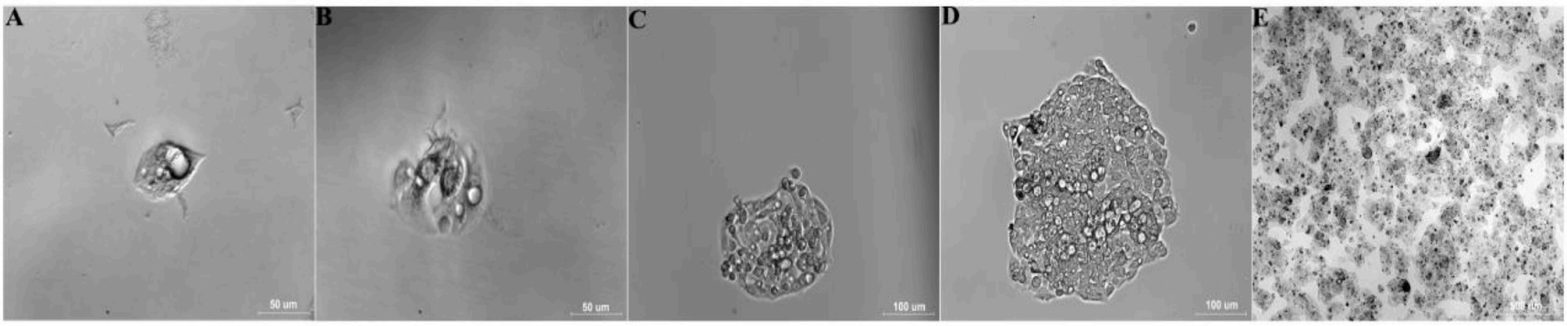
Selection and identification of monoclonal cells (Tc). Data are time after inoculation: **(A)** 12 h, **(B)** 2 d, **(C)** 5 d, **(D)** 8 d, **(E)** 27 d (25-cm^2^ tissue flask).

Four of five BALB/c mice intraperitoneally injected with 1d M(+) cells exhibited abdominal swelling after 14 days. The cells isolated from ascites of the four mice were separately inoculated in 96 well plates to select monoclonal cells. At 12 h after inoculation, single cell was detected attached to the 96 well plate, and two nuclei, representing the cell in split phase, appeared at 48 h (Figures 3A,B). By day 5, one single cell formed a cell colony with a morphology similar to normal C3A cells (Figure 3C). The single colony continued to grow and multi-layered cells were observed on day 8 which exhibited contact inhibition (Figure 3D). By day 27, the cells exhibited vigorous growth and had formed many colonies (Figure 3E).

### Successful elimination of *M. hyorhinis* confirmed by PCR and TEM

The culture supernatant from Tc and 1d M(+) cell cells was tested for *Mycoplasma* using PCR assays. The 343 bp band indicating *Mycoplasma* contamination was seen in cells from the 1d M(+) cell group, and persisted for 43 days. The Tc cells from four mice ascites were shown to be consistently *Mycoplasma* negative after 27 days of cultivation (Figure 2B).

**Figure 4:**
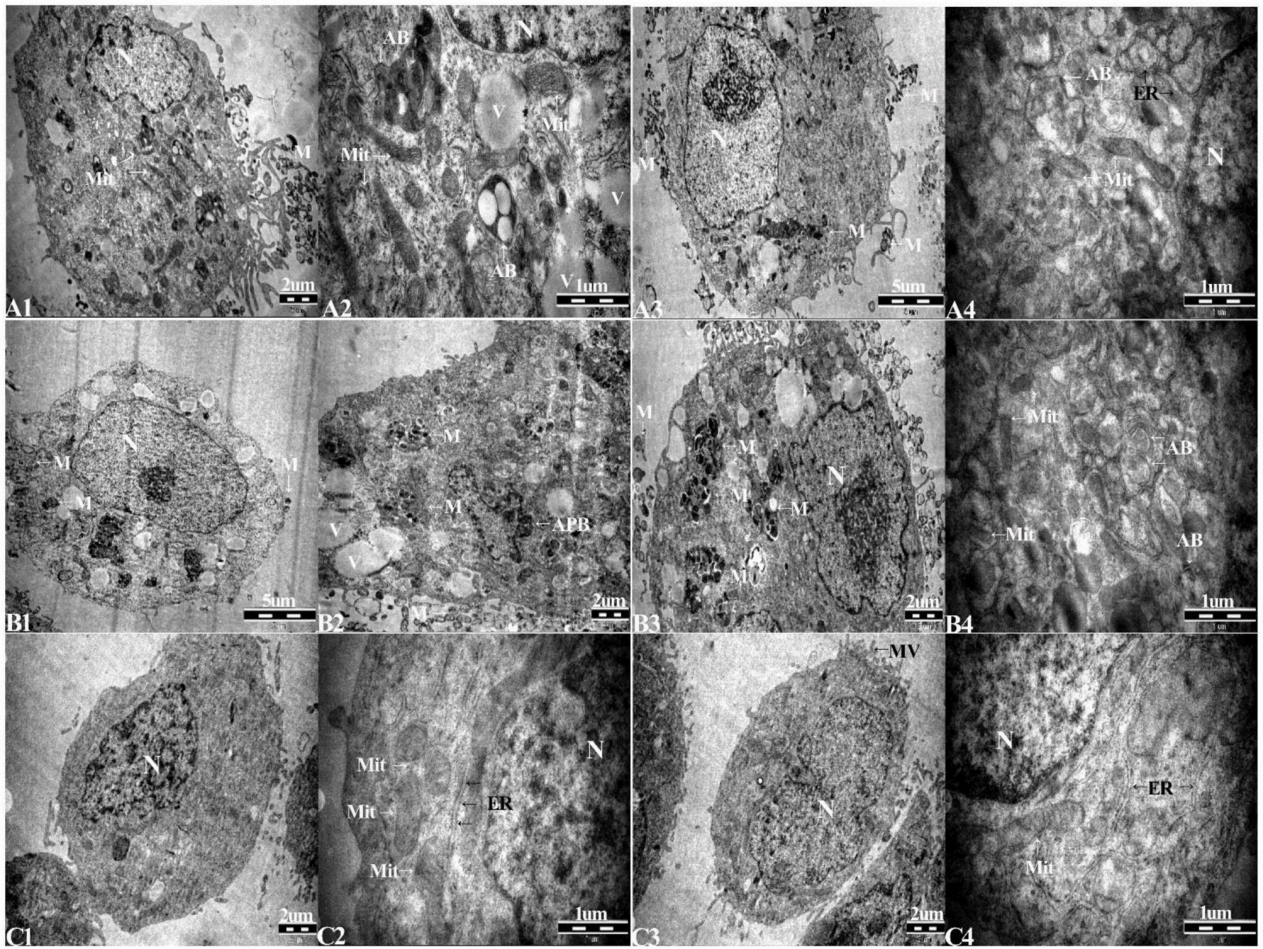
Transmission electron microscopy (TEM) of **(A1–A4)**, 1d M(+) cell; **(B1–B4)**, 43d M(+) cell; and **(C1–C4)**, Tc. *M, Mycoplasma*; AB, autophagy body; APB, apoptotic body; Mit, mitochondria; ER, endoplasmic reticulum; V, vacuoles.

TEM was used to identify *Mycoplasma* contamination and the cell state. Cells in the 1d M(+) cell group showed the presence of *Mycoplasma* around the cytomembrane, the mitochondria showed edema and swelling, intracellular autophagy bodies were detected, and the nuclei appeared swollen with clumped and marginated heterochromatin (Figures 4A1–A4). Most of the 43d M(+) cell cells exhibited the presence of intracellular *Mycoplasma*, lots of vacuoles, more severe mitochondrion swelling, and swollen nucleus compared to 1d M(+) cell, and apoptotic bodies in the cytoplasm (Figures 4 B1–B4). The presence of cell debris (CD) and nuclear fragments indicated the serious side-effects of *Mycoplasma* contamination on these cells (Supplementary Figure 2). TEM analysis showed no *Mycoplasma* either inside or outside of cells from Tc cells treated with intraperitoneal passage in four mice. Tc cells also showed rich microvilli and neatly arranged endoplasmic reticulum, indicating that these cells were not in a condition of apoptosis. Cells from the Tc group had clear *Z* lines, compact mitochondrial crista and normal intact membrane which significantly differentiated them from the contaminated cell groups (Figures 4C1–C4). At the present time, the resulting cured cells have been cultivated continuously for 18 months, during which regular screening has failed to reveal any mycoplasma infection (data not shown).

### Detection of human–mouse cross-contamination

DNA was extracted from Tc, M(−) cell, and AML12 cells for evaluate human–mouse cell cross-contamination. Human COX-1 primers generated only one band, while the mouse COX-1 primers failed to generate a band when PCR assays were performed on DNA templates from Tc cells. M(−) cells used as a human positive C3A control also showed a single 300-bp band with the human COX-I primers and no band with the mouse COX-1 primers. AML12 cells used as a mouse positive control showed a single 150-bp band with the mouse COX-I primers and no band with human COX-I primers. The negative control group was negative (Figure 2C). These results indicated that there was no contamination of the treated human cells with mouse cells.

### STR identification of Tc

**Figure 5:**
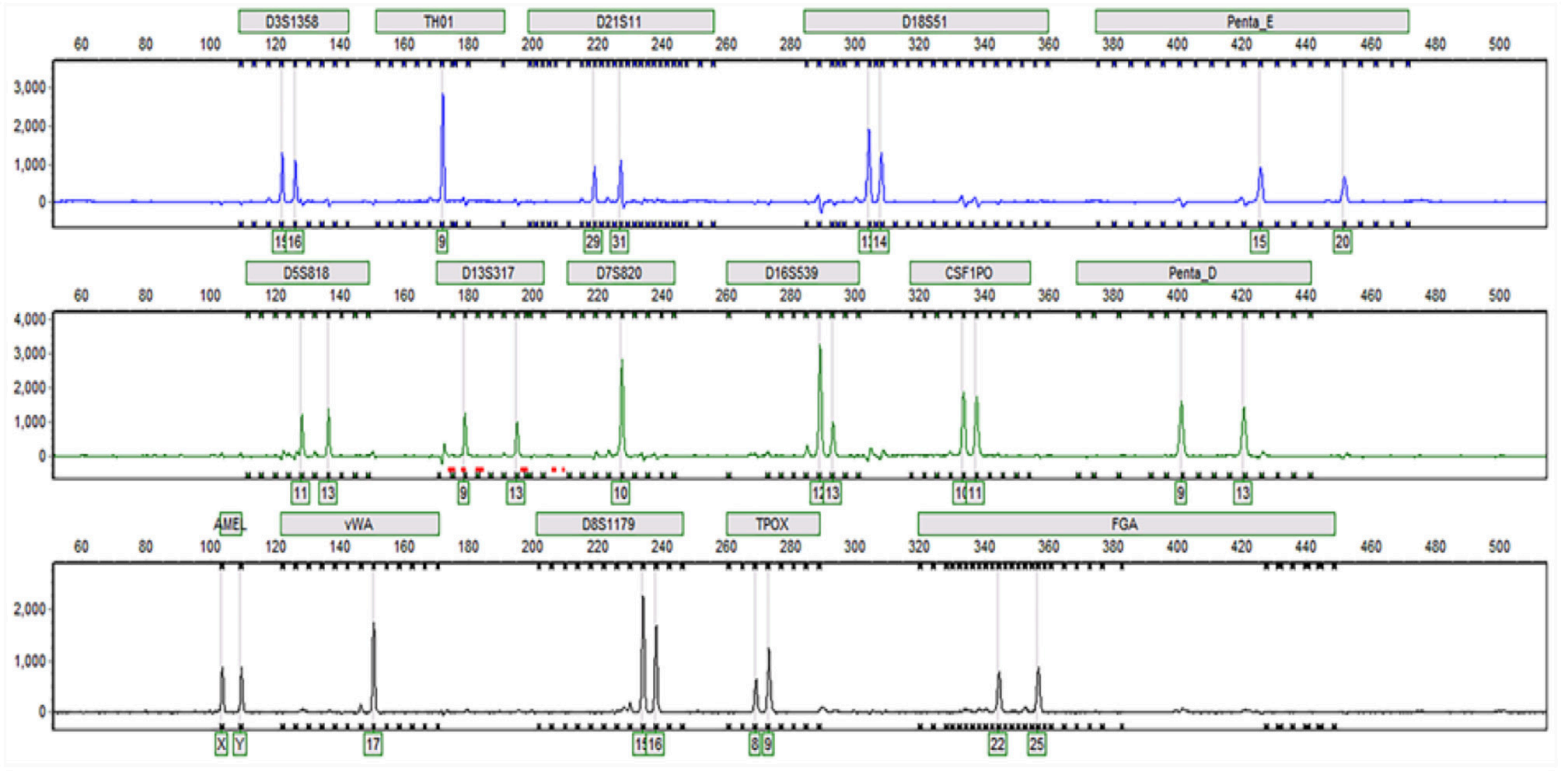
Capillary electrophoresis of Tc (D5S818, TH01, D13S317, D7S820, D16S539, CSF1PO, vWA, TPOX, Amelogenin, D3S1358, D21S11, D18S51, Penta E, Penta D, D8S1179, FGA).

Sixteen STR loci were detected by capillary electrophoresis. Eight STR loci were consistent with C3A data available from ATCC (Figure 5, Table 2). Tc cells were identified as C3A cells without contamination from mice or other species.

**Table 2:** Tc STR results and comparison.

**Locus name**	**Tc**	**C3A data**
D5S818	11, 13	11, 13
TH01	9	9
D13S317	9, 13	9, 13
D7S820	10	10
D16S539	12, 13	12, 13
CSF1PO	10, 11	10, 11
vWA	17	17
TPOX	8, 9	8, 9
Amelogenin	X, Y	X, Y
D3S1358	15, 16	–
D21S11	29, 31	–
D18S51	13, 14	–
Penta E	15, 20	–
Penta D	9, 13	–
D8S1179	15, 16	–
FGA	22, 25	–

### Hoechst 33258 staining and observation

**Figure 6:**
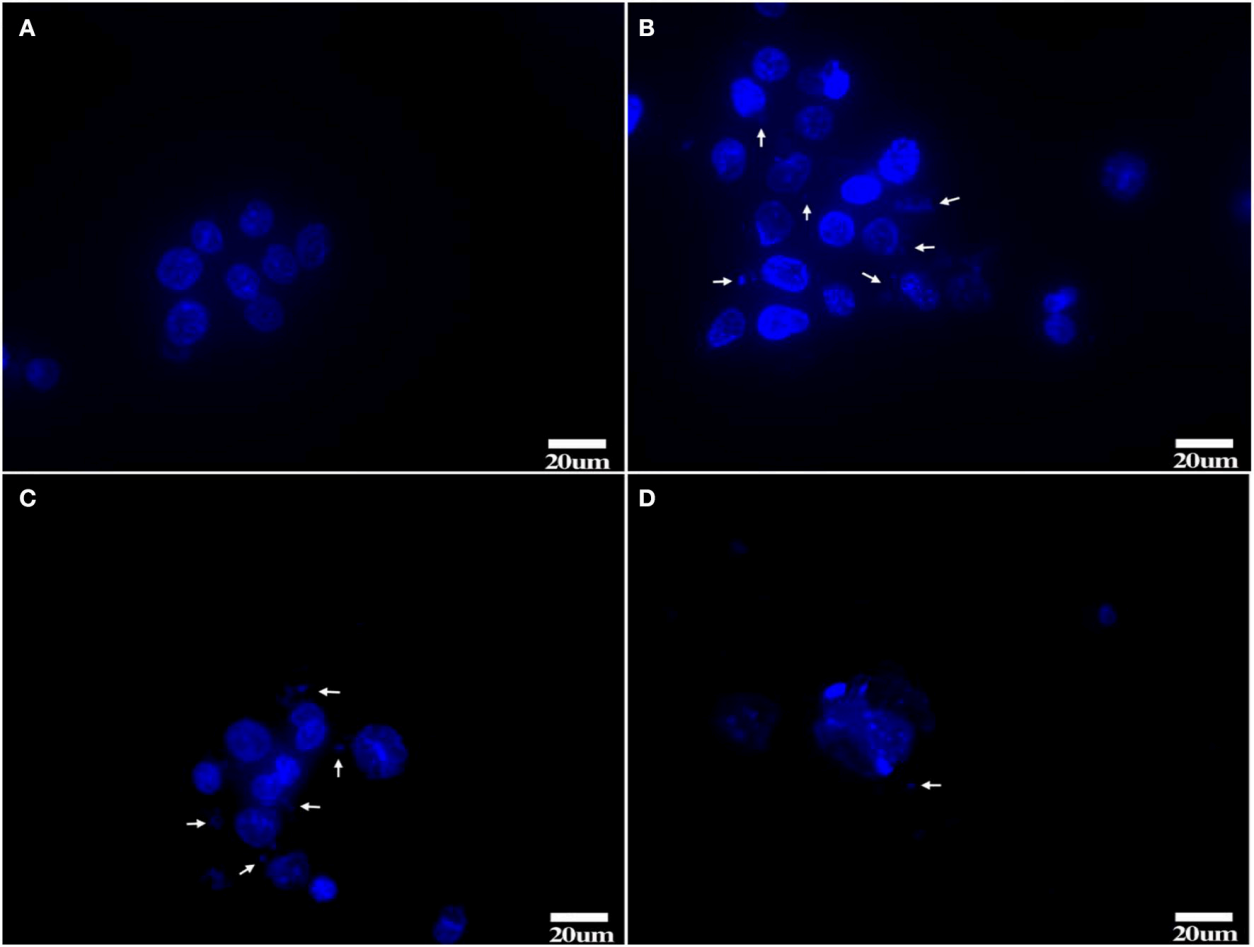
Hoechst 33258 staining **(A)** Tc, **(B,C)** 1d M(+) cell, **(D)** 43d M(+) cell. *Mycoplasma* (arrow). Tc exhibited distinct nuclei with no evidence of *Mycoplasma*; 1d M(+) cell exhibited filamentous or small granular blue fluorescence around the nuclei which were evidence of *Mycoplasma* (White arrow). 43d M(+) cell exhibited broken and fragmented nuclei with almost no intact nuclei observed. Nuclear fragmentation and intense staining was due to DNA condensation during apoptosis1d M(+) cell and 43d M(+) cell cells.

After Hoechst 33258 staining, Tc showed clear nuclei without any staining around them (Figure 6A). 1d M(+) cells showed the presence of filamentous or small granular blue fluorescence around the nuclei indicating *Mycoplasma* contamination (White arrow, Figures 6B,C). In 43d M(+) cells, there were very few intact nuclei, and most of the nuclei were broken and fragmented (Figure 6D). Nuclear fragmentation and intense nuclear staining in cells from the 1d M(+) cell and 43d M(+) cell groups indicated apoptosis.

### XMRV detection

**Figure 7:**
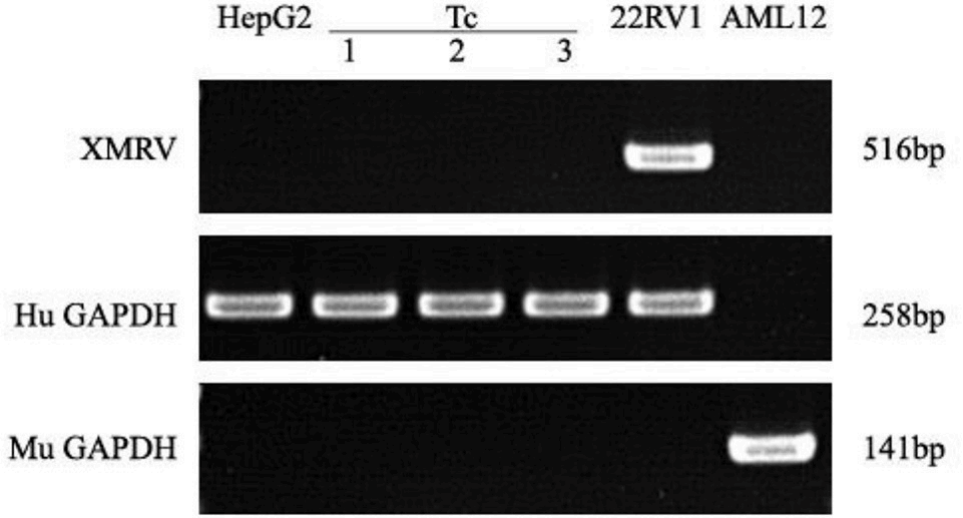
XMRV PCR detection. Tc cells were negative for XMRV expression. The positive control was 22RV1. The negative controls were AML12 and HepG2 cells. Human and Murine GAPDH gene primers served as the loading controls.

PCR assays to detect XMRV in 22Rv1 positive control human prostate cancer cells generated a band of 516 bp. PCR assays on negative control human HepG2 cells and mouse liver AML12 cells were negative (Figure 7). When DNA from Tc samples were used as a template, it was negative for the presence of XMRV.

### Elimination of mycoplasma contamination partially restored the expression of functional genes

**Figure 8:**
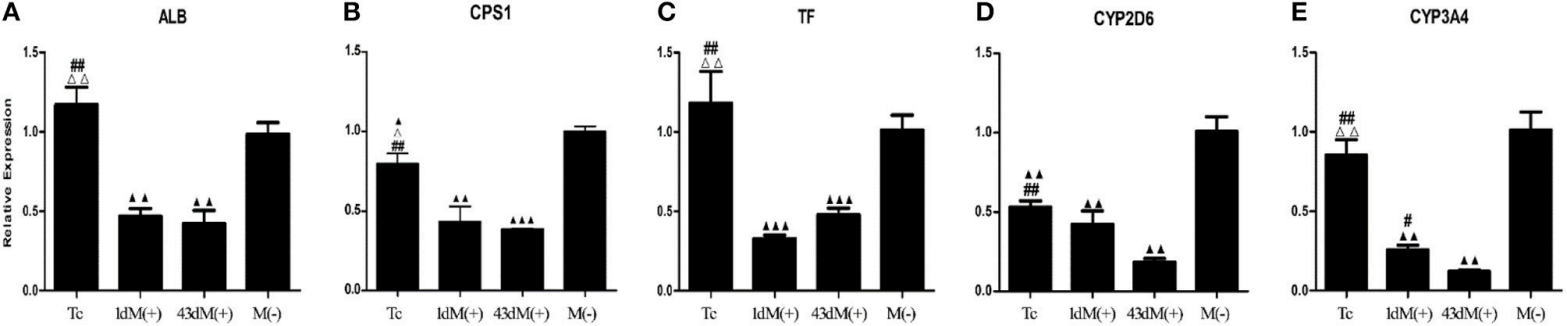
*qRT-PCR* detection of **(A)** ALB, **(B)** CPS1, **(C)** TF, **(D)** CYP3A4, **(E)** CYP2D6. Δ*P* < 0.05, ΔΔ*P* < 0.01 vs. 1d M(+) cell; #*P* < 0.05, ##*P* < 0.01 vs. 43d M(+) cell; ▲*P* < 0.05, ▲▲*P* < 0.01, ▲▲▲*P* < 0.001 vs. M(−) cell.

M(−) cell and Tc groups had almost similar expression levels of albumin (ALB), and this was significantly higher compared to 1d M(+) cell and 43d M(+) cell groups (*P* < 0.01) (Figure 8A). Carbamoyl-phosphate synthase 1 (CPS1) expression was highest in the M(−) cell group (*P* < 0.05), while Tc cells showed higher expression of CPS1 compared to the 1d M(+) cell and 43d M(+) cell groups (*P* < 0.05) (Figure 8B). Transferrin (TF) expression was significantly higher in the M(−) cell and Tc groups compared to the 1d M(+) cell and 43d M(+) cell groups (*P* < 0.01) (Figure 8C). CYP2D6 expression was significantly higher in the M(−) cell group compared to the Tc, 1d M(+) cell and 43d M(+) cell groups (*P* < 0.01), although Tc cells had a significantly higher expression compared to the 43d M(+) cell group (*P* < 0.01) (Figure 8D). Cytochrome P450 3A4 (CYP3A4) expression was significantly higher in the M(−) cell and Tc groups compared to the 1d M(+) cell and 43d M(+) cell groups (*P* < 0.01), with the 43d M(+) cell group showing the lowest level (*P* < 0.05) (Figure 8E).

### Elimination of mycoplasma contamination partially restored the expression of functional proteins

**Figure 9:**
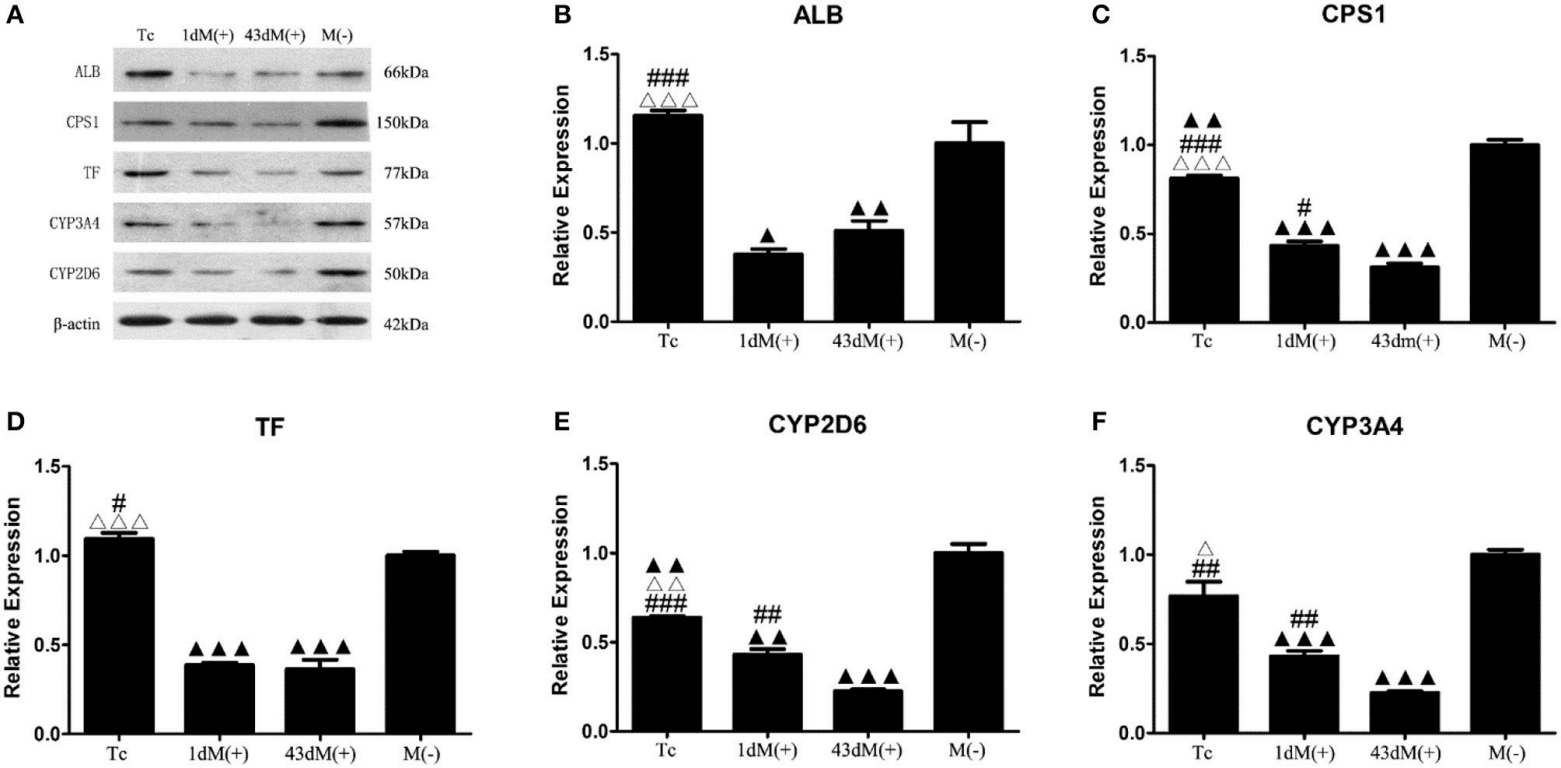
Western-Blot detection of **(A)** protein bands **(B)** ALB, **(C)** CPS1, **(D)** TF, **(E)** CYP3A4, **(F)** CYP2D6. Δ*P* < 0.05, ΔΔ*P* < 0.01, ΔΔΔ*P* < 0.001 vs. 1d M(+) cell; #*P* < 0.05, ##*P* < 0.01, ###*P* < 0.001 vs. 43d M(+) cell; ▲*P* < 0.05, ▲▲*P* < 0.01, ▲▲▲*P* < 0.001 vs. M(−) cell.

The M(−) cell and Tc groups had almost similar expression levels of ALB protein, and this was significantly higher compared to the 1d M(+) cell and 43d M(+) cell groups (*P* < 0.001) (Figures 9A,B). CPS1 protein levels were highest in the M(−) cell group (*P* < 0.01), and the Tc group had significantly higher CPS1 protein expression compared to the 1d M(+) cell and 43d M(+) cell groups (*P* < 0.001). The 1d M(+) cell group showed a significantly higher CPS1 protein expression compared to the 43d M(+) cell group (*P* < 0.05) (Figure 9C). TF protein expression was significantly higher in the M(−) cell and Tc groups compared to the 1d M(+) cell and 43d M(+) cell groups (*P* < 0.05, *P* < 0.001) (Figure 9D). CYP2D6 expression was significantly higher in the M(−) cell group compared to the Tc, M(+) cell, and 43dM(+) cell groups (*P* < 0.01), with the Tc group having significantly higher CYP2D6 levels than the 1d M(+) cell and 43d M(+) cell groups (*P* < 0.01) (Figure 9E). The M(−) cell and Tc groups had significantly higher expression of CYP3A4 protein than the 1d M(+) cell and 43d M(+) cell groups (*P* < 0.05, *P* < 0.01, *P* < 0.001), with the 43d M(+) cell group showing the lowest expression (*P* < 0.001) (Figure 9F).

### MTT assay of cell proliferation

**Figure 10:**
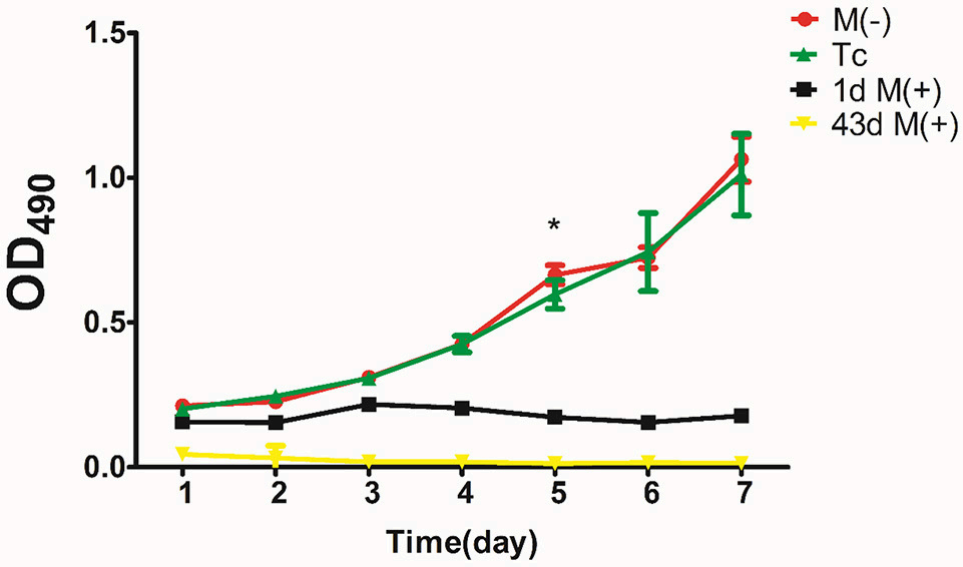
Cell proliferation evaluated by MTT assay. ^*^P < 0.001 compared with 1dM(+) cell.

There was no significant difference in proliferation between the Tc and M(−) cell groups except on day 5 (*P* < 0.05). Both groups had a significantly higher proliferation rate compared to the 1d M(+) cell and 43d M(+) cell groups (Figure 10).

## Discussion

In this study, we describe a technique to eliminate *M. hyorhinis* contamination in C3A human hepatocyte cells by intraperitoneal passage of contaminated cells in BALB/c mice. Using PCR assays with human- and mouse-specific COX-I and XMRV primers, we showed that there was no XMRV infection or cross-contamination between human and mouse cells. The decontamination was successful and lasted for more than 4 weeks without re-infection. Furthermore, the decontamination process did not compromise cell proliferation and restored the expression of important functional genes and proteins which were down-regulated in contaminated cells.

Although *Mycoplasma* infection is an insidious problem in mammalian cell culture (Gedye et al., 2015), it can be eliminated by *in vitro* and *in vivo* management. It was previously reported than an efficient way to eliminate *Mycoplasma* contamination involved intraperitoneal injection of BALB/c mice with pristanee in order to induce an inflammatory reaction and formation of ascites within 20–54 days after intraperitoneal injection with contaminated cells (Schimmelpfeng et al., 1980; Howell et al., 1982; Lombardo and Lanks, 1982; Roseto et al., 1984; Carroll and O'Kennedy, 1988; Hirschberg et al., 1989). Injection of *Mycoplasma*-contaminated cells into the abdomen of these mice resulted in elimination of *Mycoplasma*, and this was thought to be due to the activity of macrophages and cytokines released in the ascites (Roseto et al., 1984; Hirschberg et al., 1989). This was the same mechanism underlying a second previously described technique which involved the intraperitoneal injection of contaminated cell lines in nude mice without mineral oil; cells were collected by sequential washings with a syringe (Howell et al., 1982). Although the *in vivo* passage method has been validated previously, it needs a long duration (20–54 days) to form the ascitic fluid in infected mice (Schimmelpfeng et al., 1980; Howell et al., 1982; Lombardo and Lanks, 1982; Roseto et al., 1984; Carroll and O'Kennedy, 1988; Hirschberg et al., 1989). In the present study, we aimed to shorten the process of *Mycoplasma* decontamination by injecting normal BALB/c mice with paraffin oil injection prior to injecting them with *Mycoplasma*-infected cells.

A previous study (Lombardo and Lanks, 1982) reported that mycoplasma-free cultures could be obtained from ascites after 27 days *in vivo* but not after 14 days, without using paraffin oil or pristane prior to injection of cells. Using pristane as a stimulator of inflammation, Hirschberg and et al. proved that 20 days *in vivo* passage was enough for ascites collection, but a shorter time for *in vivo* passage of cells was not tested in their study (Hirschberg et al., 1989). In this study, we sought to shorten the time for *in vivo* passage by injecting the mice with paraffin oil before intraperitoneal injection with cells. Based on previous studies (Lombardo and Lanks, 1982), we considered that 14 days for *in vivo* passage was the time-frame we might challenge in this study.

We used paraffin oil instead of pristane to induce sterile inflammation prior to injection with 0.5 ml cells suspension (4.0 × 10^6^/ml cells) contaminated with 6.2 ± 2.2 × 10^8^ CFU/ml of *M. hyorhinis*. Abdominal swelling was observed, and ascites were collected 14 days in four of five mice after intraperitoneal injection with cells. The monoclonal cells from ascites were expanded, and were *Mycoplasma*-free after continuous regular detections over a period of 18 months. Successful elimination of *Mycoplasma* from C3A cells was achieved by intraperitoneal passage in four of five mice. The duration of *in vivo* passage in this study was shortened to 14 days, which was a significant time-saving advantage compared to previous similar studies.

In most previous studies, *in vivo* passage was used to treat human–murine hybridoma or murine cell lines, which made it difficult to determine cross-contamination between murine and human cells. In our present study, we investigated whether contamination of BALB/c mouse cells could be avoided during *in-vivo* passage by using a human hepatocyte cell line, C3A, for elimination testing. Since infected cells themselves are the single most important source of further contamination (Hirschberg et al., 1989), all cells were placed in separate environments with the same culture parameters. We initially used PCR and DNA sequencing to examine *Mycoplasma* contamination from C3A cells and identify the species of *Mycoplasma*. Our data showed that all three samples (days 2, 4, 6) were contaminated with *M. hyorhinis*. It has long been assumed that *Mycoplasma*s exist outside of the eukaryotic cell membrane and the pathogenesis is mediated via cytoadherence. However, *Mycoplasma fermentans, Mycoplasma suis*, and *Mycoplasma pneumoniae* have been identified intracellularly, and it is thought that the intracellular location, even for a short period, could sequester and protect *Mycoplasma* from the effect of antibiotics (Drexler and Uphoff, 2002; Felder et al., 2011; Falagan-Lotsch et al., 2015). Our TEM and immunostaining results confirmed that *M. hyorhinis* was able to grow intracellularly as well as extracellularly. These data were consistent with a previous study (Mosmann et al., 1986, 2005), and suggested that contamination with *M. hyorhinis* could result in a high risk of treatment failure with antibiotic therapy. Our present data showed that after intraperitoneal injection of BALB/c mice with contaminated cells, and from the time of seeding the monoclonal cells in plates, the culture supernatants were negative for *Mycoplasma* for a duration of 4 weeks. PCR, TEM, and Hoechst 33258 staining of Tc showed no evidence of *Mycoplasma* contamination either intracellularly or extracellularly, suggesting that our method was effective.

Although it has been shown that phagocytosis by macrophage-mediated phagocytosis could play a role in the elimination of extracellular *Mycoplasma* (Schimmelpfeng et al., 1980), the precise mechanisms underlying the elimination of intracellular *Mycoplasma* remain unclear. It has been suggested that *Mycoplasma*-infected cells could be lysed in the presence of MTM (microbial T cell mitogens) by MTM-stimulated CD8+ lymphocytes as well as by MHC class I-restricted CTL clones of defined antigen specificity (Herrmann et al., 1990). This would result in the release of intracellular *Mycoplasma* from the lysed cells, which would be susceptible to phagocytosis (Hickmandavis et al., 2001; Woolard et al., 2005). Release of cytokines such as interferon-gamma (IFN-γ), interleukin-2 (IL-2), tumor necrosis factor beta (TNF-β also known as lymphotoxin), granulocyte macrophage-colony stimulating factor (GM-CSF), and granulysin by Th1 cells and CD8+ T cells has been shown to amplify the host immune response to intracellular microbes, and enhancing cellular immunity (Mosmann et al., 1986, 2005; Mosmann and Coffman, 1989; Stenger et al., 1998; Heidegger et al., 2015; Kornspan et al., 2015). It will be interesting to further understand the mechanisms underlying *Mycoplasma* decontamination in our experimental system.

Due to concerns about human–mouse cross-contamination, we selected monoclonal cells and determined COX-I expression, which is an easy and effective way to determine contamination of cells across different species (Hebert et al., 2003; Cooper et al., 2007; Ensenberger et al., 2010). Based on our PCR results using COX-I primers, we excluded the possibility of human murine cell cross-contamination, which were further confirmed by STR detection. Our data suggested that this technique was successful in preventing murine cell contamination of treated cells.

Human cells grown as xenografts in mice have a risk of being infected with murine retroviruses, of which XMRV is a potential human pathogen (Paprotka and Pathak, 2011; Delviksfrankenberry et al., 2012; Naseer et al., 2015). Our PCR results using XMRV primers showed no evidence of XMRV infection.

*M. hyorhinis* is one of the most common species of infectious *Mycoplasma* and represents 10–40% of the *Mycoplasma* contamination in a cell culture (UKCCCR, 2000; Drexler and Uphoff, 2002; Zinöcker et al., 2011; Vande et al., 2014; Olareringeorge and Hogenesch, 2015). *M. hyorhinis* infection has been shown to degrade host cell DNA, induce malignant transformation of human prostate cells, inhibit lymphocyte proliferation, and decrease the cytostatic activity of chemotherapy drugs (Paddenberg et al., 1998; Namiki et al., 2009; Zinöcker et al., 2011; Vande et al., 2014). However, it is not clear whether *M. hyorhinis* could lead to irreversible changes in C3A cells, or whether *in vivo Mycoplasma* elimination could reverse the expression of functional genes in these cells. We compared the mRNA and protein levels of important functional genes including those involved in protein synthesis (ALB, TF), ammonia metabolism (CPS1), and drug metabolism (CYP2D6, CYP3A4) (Rothschild et al., 1988; Beutler et al., 2000; Zhou et al., 2009; Wright et al., 2011), in all the cell groups. Compared to the Tc and M(−) cell groups, the 1d M(+) cell and 43d M(+) cell groups showed a significant decrease in the expression of ALB, CPS1, TF, CYP3A4, and CYP2D6 genes as well as proteins. There was no significant difference in the gene or protein levels of ALB, or TF between the 1d M(+) cell and 43d M(+) cell groups. The expression levels of CPS1, CYP2D6 and CYP3A4 proteins were significantly lower in the 43d M(+) cell group compared to the 1d M(+) cell group, suggesting that the extent of CPS1, CYP2D6, and CYP3A4 inhibition was related to the length of infection. Additionally, since these proteins play important roles in various metabolic pathways, it is possible that there could be a further time-dependent decrease in the metabolism of *Mycoplasma*-infected cells. Elimination of *Mycoplasma* infection restored the levels of ALB, TF, and CYP3A4 genes as well as proteins to normal levels. However, the levels of CPS1 and CYP2D6 genes and proteins did not return to the levels observed in M(−) cell. These data were consistent with previous reports that showed that *Mycoplasma* infection resulted in irreversible, permanent changes in the expression of P53, RB and a number of other genes (Paddenberg et al., 1998; Zhang et al., 2006; Namiki et al., 2009; Zinöcker et al., 2011; Vande et al., 2014). Interestingly, although the expression of CPS1 and CYP2D6 in the Tc group did not reach that of the M(−) cell group, both genes and proteins were significantly up-regulated compared with the 1d M(+) cell and 43d M(+) cell groups. These data suggested that our technique did improve cell function.

MTT assays showed no significant difference in cell proliferation between the Tc and M(−) cell groups, which had higher rates of proliferation than the 1d M(+) cell and 43d M(+) cell groups. Our TEM data revealed rich microvilli, neatly arranged endoplasmic reticulum, and mitochondria with compact crista, intact membrane, and clear Z lines in the Tc group. These data were evidence of strong metabolism and protein synthesis activity in cells from the Tc group.

Compared to antibiotic treatment, our method needs more technical skills in animal handling, which is one of the limitations of this study. Another limitation was that we only tested one concentration of cells and *Mycoplasma*. The threshold of *Mycoplasma* contamination which could be removed through this system should be further examined. In addition, the functions of some genes were not 100% recovered after the treatment, and whether it was related with the irreversible cellular damage caused by *Mycoplasma* infection remained unknown. Further study is needed to investigate the reasons why some genes function could not completely recover to normal level using this method.

In summary, we describe a reliable method to eliminate *Mycoplasma* both inside and outside of culture cells without inducing cell-cross contamination or XRMV infection. Compared to the previous similar studies, the duration of intraperitoneal passage in this study was significantly shorter. This decontamination technique significantly improved the function and proliferation of treated cells, and may be used as an alternative to antibiotic treatment for *Mycoplasma* contamination in important or irreplaceable cell lines.

## Author contributions

JW and YL mainly completed this study, who contributed equally to this work. JW finished the first draft. LC gave help in the animal test. TL assisted the cell experiment. GP and CF offered help in the WB experiment. XH offered help in MTT. HL offered help in QPCR. ZJ, ZZ, and JD offered help in other experiment. QP and YG designed the experiment, provided funding and revised the paper.

### Conflict of interest statement

The authors declare that the research was conducted in the absence of any commercial or financial relationships that could be construed as a potential conflict of interest. The reviewer YB and handling Editor declared their shared affiliation.

## Supplementary material

Supplementary Figure S1 *Mycoplasma* culture number counting, **(A)** 40×; **(B)** 100×; **(C)** 200×. *M. hyorhinis* concentration was 6.2 ± 2.2 × 10^8^CFU/ml.

Supplementary Figure S2 **(A–D)** TEM of 43d M(+) cell. M, *Mycoplasma*; CD, Cell debris; N, Nuclear Fragments.
