# Supplementary figures and images for: Elimination of Mycoplasma Contamination from Infected Human Hepatocyte C3A Cells by Intraperitoneal Injection in BALB/c Mice
Source: Front Cell Infect Microbiol. 2017 Oct 12;7:440. doi: 10.3389/fcimb.2017.00440 (PMC5643414; doi:10.3389/fcimb.2017.00440)

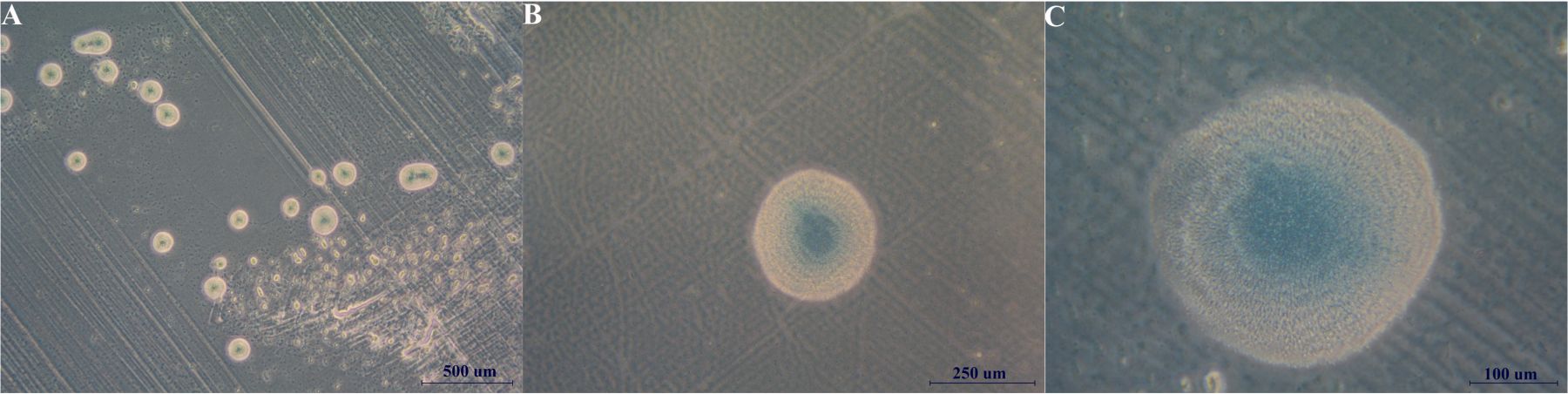

Supplement: Supplementary Figure S1 — Mycoplasma culture number counting, (A) 40×; (B) 100×; (C) 200×. M. hyorhinis concentration was 6.2 ± 2.2 × 10^8^CFU/ml. [file Image1.jpeg]

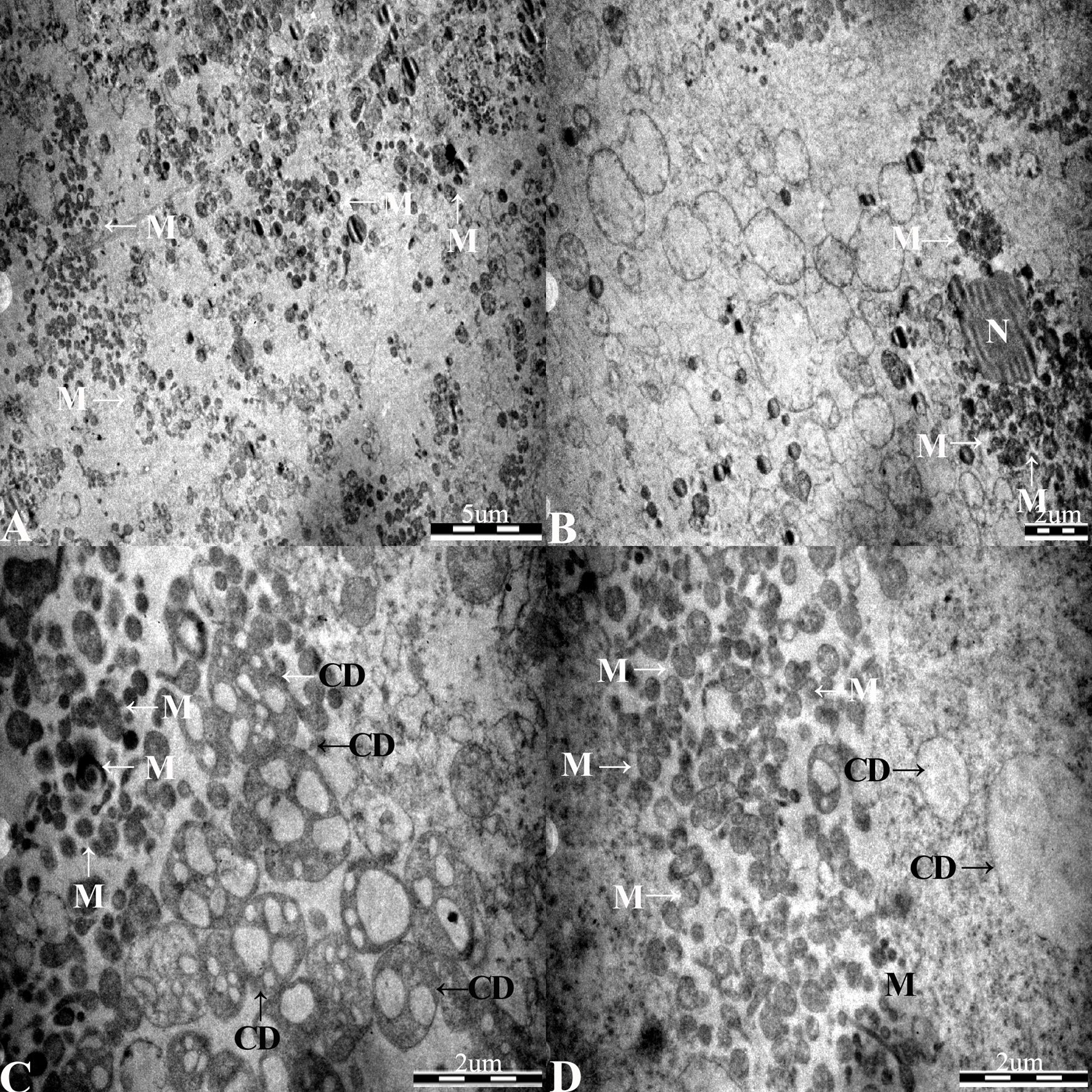

Supplement: Supplementary Figure S2 — (A–D) TEM of 43d M(+) cell. M, Mycoplasma; CD, Cell debris; N, Nuclear Fragments. [file Image2.jpeg]
